# Psychological distress among Bam earthquake survivors in Iran: a population-based study

**DOI:** 10.1186/1471-2458-5-4

**Published:** 2005-01-11

**Authors:** Ali Montazeri, Hamid Baradaran, Sepideh Omidvari, Seyed Ali Azin, Mehdi Ebadi, Gholamreza Garmaroudi, Amir Mahmood Harirchi, Mohammad Shariati

**Affiliations:** 1 Department of Social Medicine, Iranian Institute for Health Sciences Research, Tehran, Iran

## Abstract

**Background:**

An earthquake measuring 6.3 on the Richter scale struck the city of Bam in Iran on the 26th of December 2003 at 5.26 A.M. It was devastating, and left over 40,000 dead and around 30,000 injured. The profound tragedy of thousands killed has caused emotional and psychological trauma for tens of thousands of people who have survived. A study was carried out to assess psychological distress among Bam earthquake survivors and factors associated with severe mental health in those who survived the tragedy.

**Methods:**

This was a population-based study measuring psychological distress among the survivors of Bam earthquake in Iran. Using a multi-stage stratified sampling method a random sample of individuals aged 15 years and over living in Bam were interviewed. Psychological distress was measured using the 12-item General Health Questionnaire (GHQ-12).

**Results:**

In all 916 survivors were interviewed. The mean age of the respondents was 32.9 years (SD = 12.4), mostly were males (53%), married (66%) and had secondary school education (50%). Forty-one percent reported they lost 3 to 5 members of their family in the earthquake. In addition the findings showed that 58% of the respondents suffered from severe mental health as measured by the GHQ-12 and this was three times higher than reported psychological distress among the general population. There were significant differences between sub-groups of the study sample with regard to their psychological distress. The results of the logistic regression analysis also indicated that female gender; lower education, unemployment, and loss of family members were associated with severe psychological distress among earthquake victims.

**Conclusion:**

The study findings indicated that the amount of psychological distress among earthquake survivors was high and there is an urgent need to deliver mental health care to disaster victims in local medical settings and to reduce negative health impacts of the earthquake adequate psychological counseling is needed for those who survived the tragedy.

## Background

An earthquake measuring 6.3 on the Richter scale struck the city of Bam in Iran on the 26th of December 2003 at 5.26 A.M. It was devastating, the city was destroyed, left over 40,000 dead and around 30,000 injured, as well as destroying approximately 20,000 homes, leaving more than 45,000 people homeless [1]. The profound tragedy of thousands killed has caused emotional and psychological trauma for tens of thousands of people who have survived. In general earthquakes are known to be related to increased psychological symptoms among survivors and in particular it has been shown that earthquakes might cause post-traumatic stress disorder (PTSD). Several studies on psychological morbidity have been reported following earthquakes in different parts of the world [2-5], but there is no report from Iran. Iran is known to be a country of frequent severe earthquakes causing thousands of deaths in recent years. Therefore for the first time a study was conducted to measure psychological distress among the survivors of Bam earthquake in Iran. The objective was to indicate prevalence of psychological distress among survivors and to specify factors associated with psychological symptoms and thus provide information for interventions needed in Bam and in similar conditions in the country. In addition it was thought this might add to a small body of existing literature on the topic from a different population.

## Methods

This was a population-based study of psychological distress among Bam earthquake survivors aged 15 years and over at five months after the event. The pre-earthquake population of Bam was reported to be 97,000 and surprisingly it was increased to around 120,000 at the post-earthquake period (listing from the authorities). This was due to the fact that because of worry many people living in rural areas were moved to the city of Bam after earthquake. At the time of screening about 80% of people stayed inside their homes and the remaining 20% were moved to four specially designated sites with around 4000 tents. However, to select a representative sample of individuals living in Bam a stratified multi-stage area sampling was applied. The stratification was used because people living in the southern region of the city were less affluent and exposed to more damage as compared to those living in the northern part of the city. Every household or tent within the city had the same probability to be sampled. For the purposes of the first stage, units (census sections) were randomly selected after stratifying by region and size of residence. Then, within each census section the homes and tents to be sampled were selected using the procedure of random routes. Finally the last stage sampling units (the individuals) were selected randomly from all persons living in the same home or tent. Random sampling was based on a list of all households (N = 23000) derived from the electricity bills. A sample of 1150 households (5%) was selected as explained. However, we were actually able to screen 1000 households, giving a success rate of 87%. The main reason for the failed attempts for contact was due to the fact that the targeted house was uninhabited because the residents had moved out and there was no possibility to trace the survivors. In addition considering logistic difficulties we did not replace them with other households.

A team of trained interviewers collected data in a week and all participants were interviewed in their homes or designated camps. However, the data were collected in a disorganized environment and thus data gathering strategy was based on offering help to survivors. Since there was no validated measure of PTSD in the Iranian language, only psychological distress was measured using the Iranian version of the 12-item General Health Questionnaire, GHQ-12 [6]. The scale examines whether the respondent has experienced a particular symptom or behavior recently. Each item is rated on a four-point scale (less than usual, no more than usual, rather more than usual, or much more than usual). The GHQ-12 is brief, simple, easy to complete, and its application in research settings as a screening tool is well documented, and there is evidence that the GHQ-12 is a consistent and reliable instrument when used in general population samples [7]. The study used the original scoring method. In this method response categories score 0, 0, 1, and 1 respectively. This gives scores ranging from 0 to 12  and higher values indicate more psychological symptoms [8]. In addition, we collected information about demographic and the number of family members that died due to the earthquake. To analyze data Student's t-test and one-way analysis of variance were used to find out differences in psychological distress among different sub-groups of the study sample and the logistic regression analysis was performed to investigate factors associated with distress. For the purpose of the logistic regression analysis we used the population mean score on the GHQ-12 as cut-off point as recommended [9].

## Results

Data were collected from 1000 randomly selected households (600 homes and 400 tents). In total 999 survivors were interviewed, and data for 916 respondents were complete, giving a response rate of 91.7%. The mean age of the respondents was 32.9 years (SD = 12.4), and mostly were male (53%), married (66%) and had secondary school education (50%). The mean score of the survivors on the GHQ-12 was 8.7 (SD = 3.2). The findings showed that 58% of the respondents suffered from severe psychological distress as measured by the GHQ-12. Forty-one percent of the respondents reported that they lost three to five members of their family in the earthquake. The characteristics of the respondents are shown in Table 1.

**Table 1:** The characteristics of the study sample (n = 916)

		**No.**	**%**
**Age**			
	≤ 20	126	14
	21–30	352	38
	31–40	205	23
	41–50	148	16
	> 50	85	9
	Mean (SD)	32.9 (12.4)	
**Gender**			
	Male	486	53
	Female	432	47
**Marital status**			
	Single	257	28
	Married	600	66
	Divorced/widowed	59	6
**Educational level**			
	Illiterate	158	17
	Primary	181	20
	Secondary	459	50
	College/university	118	13
**Employment status**			
	Employed	233	25
	Housewife	277	30
	Student	97	11
	Unemployed	275	30
	Retired	34	4
**Number of family loss**			
	None	185	20
	< 3	261	29
	3–5	377	41
	> 5	93	10
**GHQ score**			
	Mean (SD)	8.7 (3.2)	
	< mean score	385	42
	> mean score	531	58

To examine differences in psychological distress among sub-groups of the study sample Student's t-test and one-way analysis of variance were performed. The findings showed that there were significant differences between people with different characteristics. People with old age, females, less educated individuals, divorced or widowed and unemployed respondents, and those with loss of more family members in the earthquake reported more severe psychological distress. The results are shown in Table 2.

**Table 2:** The GHQ-12 score by demographic characteristics (n = 916)

		**Mean (SD)**	**P**
**Age groups**			0.006
	≤ 20	7.8 (3.2)	
	21–30	8.6 (3.3)	
	31–40	8.9 (3.0)	
	41–50	8.9 (3.1)	
	> 50	9.3 (3.0)	
**Gender**			0.04
	Male	8.5 (3.2)	
	Female	8.9 (3.1)	
**Marital status**			< 0.0001
	Single	8.1 (3.3)	
	Married	8.9 (3.1)	
	Divorced/widowed	9.9 (2.5)	
**Educational level**			< 0.0001
	Illiterate	9.7 (2.7)	
	Primary	8.7 (3.2)	
	Secondary	8.5 (3.2)	
	College/university	8.3 (3.1)	
**Employment status**			< 0.0001
	Employed	8.4 (3.2)	
	Housewife	8.8 (3.4)	
	Student	7.0 (3.2)	
	Unemployed	9.3 (2.9)	
	Retired	9.0 (3.2)	
**Number of family loss**			< 0.0001
	None	7.7 (3.5)	
	< 3	8.7 (3.2)	
	3–5	9.1 (2.8)	
	> 5	9.3 (2.7)	

To investigate factors associated with severe psychological distress all the significant findings from the univariate analysis were entered into the logistic regression analysis. The analysis indicated that being female (OR = 2.73, 95% CI 1.19–6.26, P = 0.02), illiterate (OR = 3.36, 95% CI 1.11–10.2, P = 0.03), unemployed (OR = 4.39, 95% CI 1.56–12.4, P = 0.005), and the loss of more family members (OR for loss of more than five family members = 2.11, 95% CI 1.22–3.61, P = 0.007) were associated with more severe psychological distress. The results are shown in Table 3.

**Table 3:** The results of logistic regression analysis

	**OR (95% CI)**	**P**
**Age**	0.98 (0.95–1.01)	0.25
**Gender**		
Male	1.0 (ref.)	
Female	2.73 (1.19–6.26)	0.02
**Marital status**		
Single	1.0 (ref.)	
Married	1.56 (0.71–3.41)	0.26
Divorced/widowed	2.17 (0.50–9.37)	0.30
**Educational level**		
College/university	1.0 (ref.)	
Secondary	1.09 (0.51–2.31)	0.82
Primary	1.02 (0.43–2.43)	0.96
Illiterate	3.36 (1.11–10.2)	0.03
**Employment status**		
Housewife	1.0 (ref.)	
Employed	2.29 (0.85–6.18)	0.10
Student	1.90 (0.57–6.32)	0.29
Unemployed	4.39 (1.56–12.4)	0.005
Retired	1.43 (0.33–6.17)	0.63
**Number of family loss**		
None	1.0 (ref.)	
< 3	1.76 (1.21–2.55)	0.003
3–5	1.98 (1.32–2.98)	0.001
> 5	2.11 (1.22–3.61)	0.007

## Discussion

This was a population-based study measuring psychological distress among survivors of the Bam earthquake in Iran. Psychological morbidity was higher among females, less educated respondents and unemployed individuals. Perhaps such observation might relate to the fact that these groups of people were exposed to a relatively homogenous set of psychological stressors, leading to relatively homogenous mental health outcomes [10,11]. In addition although in the univariate analysis there were significant differences among sub-groups of the study sample with regard to their GHQ-12 mean scores, in the logistic regression analysis age and marital status no longer remained significant. This is consistent with other research findings where female gender, lower education, and lower socio-economic status were found to be related to higher PTSD and depression among earthquake survivors [12,13]. However, recent findings on the topic showed a differential predictor pattern for PTSD and depression among earthquake survivors indicating that although certain factors (e.g. grater fear during the earthquake and female gender) relate to PTSD, lower education and loss of family members tend to relate to depression and not to PTSD [13,14]. This suggests that when interpreting the study results one might relate female gender to possible PTSD which we did not measure, and the other variables to depression which was measured using the GHQ-12.

Most notably it was found that people who lost family members reported significant severe psychological distress compared to those who did not. Also there was a distinct pattern of psychological distress among those who lost their family members in the earthquake showing a dose-response relationship between loss and the risk of more psychological distress. One might argue that the relationship between loss of relatives and psychological distress may be a reflection of the relationship between severity of trauma exposure and psychopathology. Since no other variables reflecting trauma-exposure (e.g. fear during the earthquake, level of damage to home, injury, etc.) were entered in the regression equation, 'loss of relatives' was the only variable tapping that, as there is high correlation between loss of first degree relatives (with whom survivors often share the same roof) and exposure to serious damage or collapse of the house. In addition studies have indicated that loss is a strong determinant of PTSD among earthquake survivors and thus it is argued the observation that the risk of PTSD is linked to the amount of loss is an important issue that needs to be incorporated in the development of any effective preventive strategy [15]. As discussed earlier such finding has been challenged and studies have shown that PTSD and depression are distinct issues and loss only relates to depression whereas PTSD is linked to factors relating to exposure to threat or fear for life [14]. However, since in the present study only the GHQ-12 was used, there is no way of knowing which factors related to PTSD and which to depression. The GHQ-12 has items tapping depression but little is known as to how sensitive is to picking up PTSD.

Psychological distress among earthquake survivors alongside experience of other problems could be considered a serious issue for people's health status living in such difficult conditions. Evidence suggests that severe earthquakes even can cause long-standing morbidity [12]. However, past psychiatric illness also might contribute to this situation [15,16]. Unfortunately one of the shortcomings of the present study was that we did not measure previous psychiatric conditions among survivors and thus it was not possible to comment on this further.

It is recommended that the mean GHQ score for the whole population of respondents provide a rough guide to the best cut-off threshold [9]. Thus considering people who scored above the mean, the findings from the present study indicated that 58% of the respondents showed an indication of severe mental health problems. Comparing the figure with the national data this was found to be three times higher than reported mental disorders in the general population [17]. A study using the GHQ-28 measuring prevalence of psychiatric disorder following the China earthquake showed relatively a similar result where the rate of psychological morbidity for earthquake survivors was found to be 51% [18]. Thus it is argued that there is an urgent need to deliver mental health care to disaster victims in local medical settings and health care professionals who work with the earthquake victims need to be promptly and efficiently trained in mental health crisis interventions [19].

The results reported here is an estimate for the overall population experiencing the earthquake. Studies have shown that there is variation in psychological morbidity among earthquake survivors by epicenter proximity and rate of property damage [12,18]. However one should be aware that these variables are not the only information that is needed for studying the relationship between psychological distress and the impact of the earthquake. There is also need to obtain information from earthquake survivors on the extent of damage to their homes, experience of being buried under rubble, participation in rescue operations, and witnessing grotesque sites [e.g. see [13,14]].

The present study had certain methodological limitations. Firstly, the results were based on the GHQ-12 scores and we did not measure PTSD symptoms. In this respect also it is important to acknowledge that the GHQ is a general measure of mental health and it is not a measure of diagnostic depression. Thus, since depression is also common in earthquake survivors, our study is limited in not including a validated diagnostic measure of depression. Secondly, no information was obtained on important trauma-exposure variables such as extent of fear or perceived life-threat during the earthquake, rubble experience, disability or injury, et cetera. Thirdly, data on important demographic variables such as past psychiatric illness, and family psychiatric illness were not collected. However in spite of these limitations, the results from this study are useful. This study is the only paper addressing reports on the psychological consequences of the most disastrous earthquakes of the last 50 years. In addition, to our knowledge the international literature does not contain any study on the psychological status of Iranian earthquake survivors, despite the fact that Iran is an earthquake-prone country. It seems that the future research should carefully assess the psychological distress and disruption experiences of the survivors in order to implement necessary interventions. Given that Iran is a country that suffers catastrophic earthquakes relatively frequently, there is need to develop and validate standard instruments such as PTSD measures to include in the future research.

## Conclusions

In conclusion the findings from this study indicated that the survivors of Bam earthquake suffer from psychological distress three times higher than the normal population. In addition female gender, lower education, unemployment, and loss of family members were found to be associated with more severe psychological morbidity among survivors. This suggests that to reduce negative health impacts of the earthquake adequate psychological counseling is needed for those who survived the tragedy.

## Abbreviations

GHQ-12: The 12-item General Health Questionnaire; PTSD: Post-traumatic stress disorder; SD: Standard deviation; OR: Odds ratio, CI: Confidence interval.

## Competing interests

The author(s) declare that they have no competing interests.

## Authors' contributions

AM was the principle investigator and contributed to the study design, the data analysis and wrote the paper. All other investigators contributed to the study design, the data collection and analysis. All authors reviewed and contributed to the final manuscript.

## Pre-publication history

The pre-publication history for this paper can be accessed here:



## References

[B1] World Health Organization (2004). WHO joins international effort to help Bam earthquake survivors. Bulletin of WHO.

[B2] Basoglu M, Salciooglu E, Livanou M (2002). Traumatic stress responses in earthquake survivors in Turky. J Trauma Stress.

[B3] Scott RL, Knoth RL, Beltran-Quiones M, Gomez N (2003). Assessment of psychological functioning in adolescent earthquake victims in Colombia using the MMPI-A. J Trauma Stress.

[B4] Lai TJ, Chang CM, Connor KM, Lee LC, Davidson JR (2004). Full and partial PTSD among earthquake survivors in rural Taiwan. J Psychiatr Res.

[B5] Bodvarsdottir I, Elklit A (2004). Psychological reactions in Icelandic earthquake survivors. Scand J Psychol.

[B6] Montazeri A, Harirchi AM, Shariati M, Garmaroudi Gh, Ebadi M, Fateh A (2003). The 12-item General Health Questionnaire (GHQ-12): translation and validation study of the Iranian version. Health and Quality of Life Outcomes.

[B7] Pevalin DJ (2000). Multiple applications of the GHQ-12 in a general population sample: an investigation of long-term retest effects. Soc Psychiatry Psychiatr Epidemiol.

[B8] Goldberg D (1992). General Health Questionnaire (GHQ-12).

[B9] Goldberg DP, Oldhinkel T, Ormel J (1998). Why GHQ threshold varies from one place to another. Psychol Med.

[B10] Krieger N (2001). Theories for social epidemiology in the 21^st ^century: an eco-social perspective. Int J Epidemiol.

[B11] Ferrer RL, Palmer R (2004). Variations in health status within and between socioeconomic strata. J Epidemiol Community Health.

[B12] Kilic C, Ulusoy M (2003). Psychological effects of the November 1999 earthquake in Turkey: an epidemiological study. Acta Psychiatr Scand.

[B13] Livanou M, Basoglu M, Salcioglu E, Kalendar D (2002). Traumatic stress responses in treatment-seeking earthquake survivors in Turkey. J Nerv Ment Dis.

[B14] Basoglu M, Kilic C, Salcioglu E, Livanou M (2004). Prevalence of posttraumatic stress disorder and comorbid depression in earthquake survivors in Turkey: an epidemiological study. J Trauma Stress.

[B15] Armenian HK, Morikawa M, Melkonian AK (2000). Loss as a determinant of PTSD in a cohort of adult survivors of the 1988 earthquake in Armenia: implications for policy. Acta Psychiatr Scand.

[B16] Wang X, Gao L, Shinfuku N, Zhang H, Zhao C, Shen Y (2000). Longitudinal study of earthquake related PTSD in a randomly selected community sample in North China. Am J Psychiatry.

[B17] Noorbala AA, Bagheri Yazdi SA, Yasamy MT, Mohammad K (2004). Mental health survey of the adult population in Iran. Br J Psychiatry.

[B18] Cao H, McFarlane AC, Klimidis S (2003). Prevalence of psychiatric disorder following the 1988 Yun Nan (China) earthquake. The first 5-month period. Soc Psychiatry Psychiatr Epidemiol.

[B19] Yang YK, Yeh TL, Chen CC, Lee CK, Lee IH, Lee LC, Jeffries KJ (2003). Psychiatric morbidity and posttraumatic symptoms among earthquake victims in primary care clinics. Gen Hosp Psychiatry.

